# Benzoylated Uronic Acid Building Blocks and Synthesis of *N*-Uronate Conjugates of Lamotrigine

**DOI:** 10.3390/molecules17010820

**Published:** 2012-01-16

**Authors:** Aslan M. Esmurziev, Arne Reimers, Trygve Andreassen, Nebojsa Simic, Eirik Sundby, Bård Helge Hoff

**Affiliations:** 1 Norwegian University of Science and Technology (NTNU), Høgskoleringen 5, Trondheim NO-7491, Norway; 2 Sør-Trøndelag University College, E.C. Dahls Gate 2, Trondheim NO-7004, Norway; 3 Department of Neuroscience, Faculty of Medicine, Norwegian University of Science and Technology (NTNU), Trondheim NO-7491, Norway; 4 Department of Biotechnology, Norwegian University of Science and Technology (NTNU), Trondheim NO-7491, Norway

**Keywords:** uronic acid building blocks, lamotrigine-N2-glucuronide, fluorinated carbohydrates

## Abstract

A chemoenzymatic approach towards benzoylated uronic acid building blocks has been investigated starting with benzoylated hexapyranosides using regioselective C-6 enzymatic hydrolysis as the key step. Two of the building blocks were reacted with the antiepileptic drug lamotrigine. Glucuronidation of lamotrigine using methyl (2,3,4-tri-*O*-benzoyl-α-d-glycopyranosyl bromide)uronate proceeded to give the N2-conjugate. However, lamotrigine-N2-glucuronide was most efficiently synthesised from methyl (2,3,4-tri-*O*-acetyl-α-d-glucopyranosyl bromide)uronate. Employing nitromethane as solvent with CdCO_3_ as a base lamotrigine-N2 glucuronide was prepared in a high yield (41%). Also methyl (2,3-di-*O*-benzoyl-4-deoxy-4-fluoro-α-d-glucosyl bromide)uronate underwent *N*-glucuronidation, but the product was unstable, eliminating hydrogen fluoride to give the corresponding enoate conjugate.

## 1. Introduction

Uronic acid derivatives are useful building blocks for preparing, among others, synthetic glycoproteins [1], proteoglycans [2,3], functional polymers [4,5], and artificial carbohydrates by reaction at C-6 [6,7]. They have also been suggested as components in prodrugs [8,9] and biologically active compounds [10,11]. Fluorinated uronic acid derivatives have previously been used in elucidation of enzymatic mechanisms [12,13,14,15]. However, most importantly, d-glucuronic acid is a building block for preparing *S, N* and *O*-glucuronides [16,17,18], the metabolic products of human detoxification.

Lamotrigine (LTG) is a so-called second-generation antiepileptic drug that is being used in a variety of epileptic syndromes as well as in bipolar disorder. LTG is metabolized primarily by conjugation with glucuronic acid, mainly by uridine 5′-diphospho-glucuronosyltransferase 1A4 (UGT-1A4), and presumably also by UGT-2B7 [19,20]. Approximately 70% of an oral dose of LTG is found in the urine as the corresponding N2-glucuronide. A N5-glucuronide has been postulated [21], but has, to our knowledge, never been demonstrated. LTG is subject to marked pharmacokinetic drug-drug interactions with enzyme-inhibiting and -inducing drugs. Moreover, its serum concentrations decline considerably during pregnancy [22]. To investigate the underlying mechanisms for these observations, it is desirable not only to quantify the parent compound, but also the main metabolite. On this background we have undertaken the synthesis uronic acid building blocks and have tested their use in preparation of LTG-uronic acid conjugates.

## 2. Results and Discussion

### 2.1. Uronic Acid Building Blocks

**Scheme 1:**
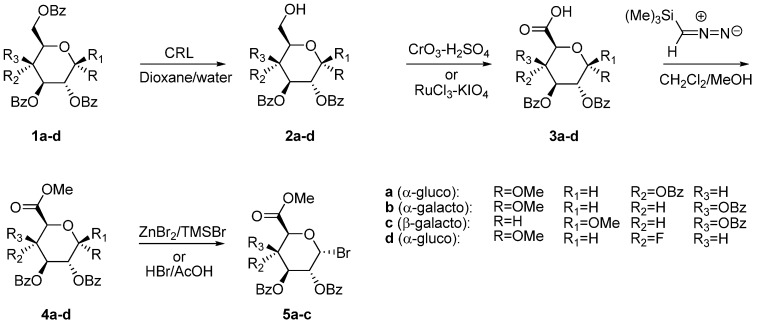
Synthetic route to the uronic acid building blocks.

To obtain benzoylated uronic acid derivatives the route shown in Scheme 1 was employed. The starting materials **1a–c** were prepared by standard benzoylation [23], whereas **1d** was made by fluorination of methyl 2,3,6-tri-*O*-benzoyl α-d-galactopyranoside using DAST or Deoxo-Fluor^TM^ [24]. Enzymatic hydrolysis with *Candida rugosa* lipase in dioxane/water afforded a regioselective debenzoylation at position 6 giving compounds **2a–d**.

**Scheme 2:**
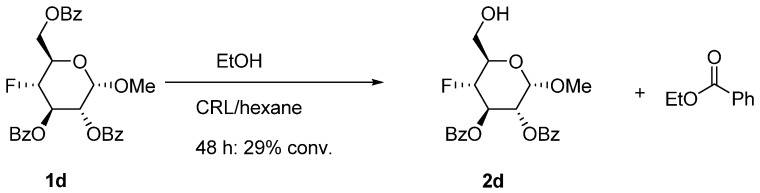
Ethanolysis of **1d** using *Candida rugosa*.

In CRL catalysed hydrolysis of the 4-deoxy-4-fluoro derivative **1d**, inhibition phenomena where encountered and full conversion was not reached by a standard single run experiment or by the addition of extra fresh enzyme during the reaction. However, by performing an extractive work-up and restarting the hydrolysis, 90% conversion and 76% isolated yield of **2d** was obtained in gram scale synthesis. Ethanolysis of **1d** to **2d** in hexane was also tested (Scheme 2). A 29% conversion (23% isolated yield) was obtained after 48 h. reaction time. Due to the low rate of reaction this strategy was not investigated further. No benzoyl migration was observed for these compounds under storage as solids or in chloroform solution.

Oxidations of carbohydrates can be performed regioselectively using laccases and TEMPO. In our hands, the model compound phenyl α*-*d-glucoside was efficiently converted to the corresponding acid. However, no reaction could be observed when **2a** was subjected to the same reaction conditions, possibly due to insolubility of the substrate and phase transfer limitations. We therefore turned our attention to alternative oxidation systems. Employing RuCl_3_ and potassium periodate as oxidant [25] in a water/acetone solvent mixture, only trace amount of product was observed after 48 h. Changing the solvent to a mixture of acetonitrile, water and carbon tetrachloride proved successful and full conversion was obtained in 4 h reaction time. A Jones oxidation [26], with CrO_3_-H_2_SO_4_ was also slow when performed in acetone or in acetonitrile/water/CCl_4_ mixture. However, when performed in acetonitrile/CCl_4_, full conversion was obtained in 6–7 h. By this method the uronic acids **3a–d** were obtained in 74–79% isolated yield. 

Methylation of the carboxylic acid function was performed using trimethylsilyl diazomethane. In pure dichloromethane the reaction did not proceed to completion. However, by applying methanol as a co-solvent full conversion was obtained in 45 min at 0 °C. This gave the uronic acid methyl esters **4a–d** in 73–82% isolated yield after crystallisation.

Functional group interconversion of the methyl acetal function to the corresponding bromide was performed by two methods. In a small 50 mg scale, **4a–c** could be reacted with HBr/acetic acid to give the 1-bromo compounds **5a–c** in 44–59% yield after crystallisation. The anomeric configuration was independent of the configuration of the starting material. Thus, both α-anomer **4b** and β anomer **4c** upon bromination gave **5b**, in accordance with the high preference for axial positioning of the bromo substituents at C-1 [27,28]. Reactions at a 0.5 g scale proceeded more slowly and more impurities were observed, which complicated purification by crystallisation. Silica gel column chromatography resulted in decomposition of **5a-d** and a low yield. Therefore, zinc bromide in combination with trimethylsilane bromide [29], was tested in brominations of **4a** and **4d**. The reaction proceeded to give **5a** and **5d** as sole products, and allowed for the isolation of **5a** and **5d** in 75 and 77% yield by crystallisation from ethanol.

**Scheme 3:**
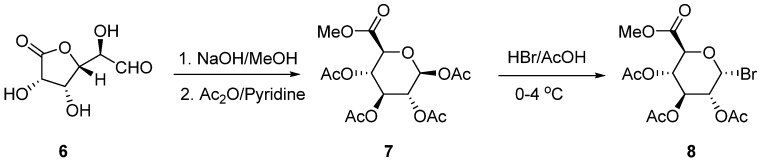
Synthesis of methyl (2,3,4-tri-*O*-acetyl-α-d-glucopyranosyl bromide)uronate.

As an alternative building block for preparation of the N-2-glucuronide of lamotrigine, the acetylated glucuronic acid derivative **8** was made from d-glucuronolactone (**6**) by methylation, acetylation and bromination (Scheme 3). Using the method as described by Caldwell *et al.* [30] more than 30 g of **8** was synthesised starting from 50 g of **6**.

### 2.2. Uronic Acid Conjugates

**Scheme 4:**
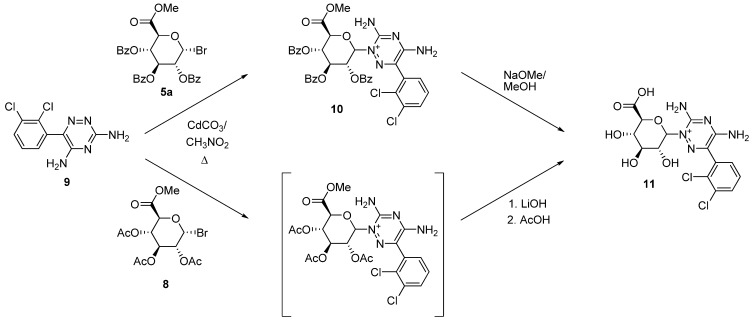
Routes tested for preparation of the *N*-glucuronate **11**.

Quaternary *N*-glucuronides of 1-phenylimidazole [31], cotinine [30], and other nicotine derivatives [32], have previously been synthesised using methyl (2,3,4-tri-*O*-acetyl-α-d-glucopyranosyl bromide) uronate (**8**) in melt form without a base. Using compound **8** as starting material in reaction with lamotrigine (**9)**, only a low 8% of the *N*-glucuronide **11** was obtained by this method. Instead, reactions with CdCO_3_, previously reported to be an efficient base in *N*-glycosylations, were tested on both **5a** and **8** [33,34,35]. In both cases a rapid conversion towards the product was evident when the coupling was performed in refluxing nitromethane (Scheme 4). Starting with **8**, hydrolysis of the acetyl functions was performed without isolation of the intermediate using lithium hydroxide followed by pH adjustment with AcOH. The lyophilized product was first purified by cation exchange to a purity of 95%. A final purification by preparative HPLC using a SB-C18 column resulted in a purity of >99% (HPLC-UV) in 41% yield. This compares very favourably with all previous reported synthesis of quaternary *N*-glucuronides. The reason for the conversion improvement upon using CdCO_3_ is not firmly established. However, it has been reasoned that the *in situ* formation of a Cd^2+^ halide is responsible for a heterogeneous catalysis involving these speices [33].

Starting with the benzoylated **5a** an 80% conversion to **10** was obtained in 3 h. Conversion could not be improved by extending the reaction time. Following extractive work-up, preparative TLC and crystallisation, 51% of a material indicated by NMR and MS to be a mixture of the α- and β-anomer of **10**. Methanolysis of **10** gave the corresponding *N*-glucuronide **11**, but we were not able to purify this material as efficiently as when **8** was used as starting material. Possibly, the challenges encountered with **5a** as a building block in this reaction are related to a high anomerisation rate, and the presence of the open form of the hexose as observed for other benzoylated uronic acid derivatives [36].

**Scheme 5:**
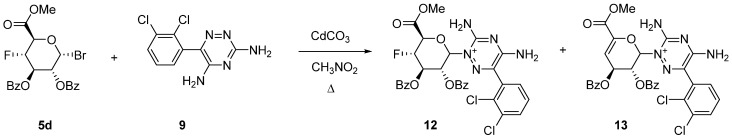
Synthesis of the *N*-glucuronate derivatives **12** and **13**.

In analysis of drug metabolites such as glucuronide conjugates, structurally related standards would be highly desirable. The 4-deoxy-4-fluoroglucuronide might in this respect be useful. An alternative starting material is **5d**, which was reacted with lamotrigine (**9**) as shown in Scheme 5.

The reaction gave, according to MS and NMR the *N*-glucuronate **12**, however, the product proved unstable under the reaction conditions, and the defluorinated enoate **13** was also formed. The ratio of **12**/**13** depended on degree of conversion, and a higher amount of **12** was observed when lowering the amount of base. However, these conditions gave low overall conversion and did not allow for efficient isolation of the synthesised products. By employing refluxing conditions and short reaction time, **12** and **13** were isolated semipure in 16% and 13% yield respectively. Elimination reactions have also previously been reported for various galacto derivatives [37,38,39,40], and given the challenges also with non fluorinated analouges, this strategy was discontinued. Rhy *et al.* [12] has prepared disaccharides containing a 4-fluorinated uronic acid, by performing 6-C oxidation after glycoside coupling. Provided mild conditions could be identified, this might be an alternative strategy to such fluorinated glucuronides.

### 2.3. NMR Characterisation of Lamotrigin Glucuronide

The LTG-glucuronide **11** was subjected to high field NMR analysis. The chemical shift values are summarized in Table 1.

## 3. Experimental

### 3.1. Chemicals and Equipment

*Candida rugosa* lipase was from Sigma-Aldrich (type VII, ≥700 units/mg solid). Compounds **1a–c** [23], **1d** [24], **2a–c** [41] were prepared as described previously. Silica gel column chromatography was performed using silica gel 60A from Fluka, pore size 40–63 μm. Accurate mass determination (ESI) was performed on an Agilent G1969 TOF MS instrument equipped with a dual electrospray ion source. Melting points were measured by a Mettler FP 5 melting point apparatus and are uncorrected. Optical rotations were measured using sodium D line at 589 nm on a Perkin-Elmer 243 B polarimeter. The HPLC system consisted of an Agilent 1100 series quaternary pump, Agilent 1100 series variable wavelength UV-detector (200–315 nm) and a thermostated column compartment. For all analysis samples were analysed on a Supelcosil C18 column [250 × 4.6 mm, 5 μm, 65:35 hexanesulfonic acid (0.05 M)-MeOH, flow 1 mL/min]. Preparative HPLC was conducted on a Agilent SB-C18 column [10 × 50 mm, 5 μm, methanol with 0.05% HCOOH-H_2_O (1:3), flow 4 mL/min] using repeated injections. UV detection was set to wavelength 254 nm for all samples.

### 3.2. NMR Spectroscopy

^1^H- and ^13^C-NMR spectra were recorded with Bruker Avance 400 or 600 spectrometers. ^19^F-NMR was performed on a Bruker Avance 500 operating at 470 MHz. For ^1^H- and ^13^C-NMR chemical shifts are in ppm rel. to TMS, while for ^19^F-NMR the shift values are relative to hexafluorobenzene. Coupling constants are in Hertz.

Compound **11**: ^1^H- and ^13^C-NMR spectra were recorded and assigned by using IP-COSY, NOESY, HSQC and HMBC experiments. ^1^H-NMR, ^13^C-NMR, NOESY and HSQC spectra were recorded on a Bruker Avance 600 FT-NMR Spectrometer, equipped with a TCI CryoProbe. IP-COSY and HMBC spectra were recorded on a Bruker DPX 400 FT-NMR Spectrometer equipped with a PADUL probe. The NMR solvent (for LTG-glucuronide) used was H_2_O added 10% D_2_O to provide a lock signal. Acetone was used as internal standard (^1^H shift 2.218 ppm, ^13^C shift 30.89 ppm). For the ^1^H-NMR, IP-COSY and NOESY experiments, excitation sculpting was used to suppress the water signal. For all experiments, the number of transients was varied to obtain the required signal-to-noise ratio. IP-COSY data were acquired as 2,048 × 128 complex points with spectral widths of 6410 Hz for both frequency domains and 1.5 s relaxation delay. The NOESY data were acquired as 2048 × 256 complex points with spectral widths of 6002 Hz for both frequency domains, a mixing time of 500 ms and 1.0 s relaxation delay. The HSQC data were acquired as 2,048 × 256 complex points with spectral widths of 9,615 Hz and 24,148 Hz in F_2_ and F_1_ respectively. The experiment had a relaxation delay of 1.0 s and was optimized for an assumed direct coupling of 145 Hz. The HMBC data were acquired as 1,024 × 256 data points with spectral widths of 4,006 Hz and 20,124 Hz in F_2_ and F_1_ respectively. This experiment has a relaxation delay of 0.9 s and was optimized for an assumed long-range coupling of 8 Hz.

### 3.3. Methyl 2,3-di-O-Benzoyl-4-deoxy-4-fluoro-α-d-glucopyranoside *(**2d**)*

*Methyl 2,3,6-tri-*O*-benzoyl-4-deoxy-4-fluoro-α-d-glucopyranoside* (**1d**, 3.00 g, 5.90 mmol) was suspended in 20% dioxane/acetate buffer at pH 4.8 (432 mL). Lipase from *Candida rugosa* (1.5 g) was added, and the mixture was agitated (200 rpm) at 30 °C for 48 h. Then the reaction mixture was filtered through Celite, followed by washing with EtOAc (200 mL) and the organic phase was washed with saturated NaHCO_3_ (3 × 300 mL) and water (3 × 300 mL). The water fraction was back extracted with EtOAc (2 × 500 mL). After drying over MgSO_4_ and evaporation, the resulting syrup was treated once more under identical conditions. Purification by silica gel column chromatography (toluene/EtOAc, 1/1) and crystallisation from diethyl ether/hexane gave 1.82 g (4.50 mmol, 76%) of a white solid, mp. 111–112 °C, [α]${}_{D}^{20}$: = +127.0 (c 1.00, CHCl_3_). ^1^H-NMR (400 MHz, CDCl_3_) δ: 7.99 (m, 4H), 7.50 (m, 2H), 7.37 (m, 4H), 6.09 (m, 1H, H-3), 5.16 (t, *J* = 3.4, 1H, H-1), 5.12 (dd, *J* = 10.2, 3.4, 1H, H-2), 4.78 (dt, *J* = 50.5, 9.2, H-4), 3.98 (m, 2H, H-5+H-6_a_), 3.89 (m, 1H, H6_b_), 3.44 (s, 3H). ^13^C-NMR (100 MHz, CDCl_3_) δ: 166.0, 165.8, 133.6–128.4 (12C), 97.1 (C-1), 87.7 (d, *J* = 185.6, C-4), 71.7 (d, *J* = 8.1, C-2), 70.6 (d, *J* = 20.2, C-3), 69.2 (d, *J* = 24.3, H-5), 61.0 (C-6), 55.8 (OMe).^19^F-NMR (470 MHz, CDCl_3_) δ: −198.26 (dd, *J* = 50.5, 13.8). HRMS (ESI): 405.1337 (calcd. 405.1344, M+H^+^). 

### 3.4. Oxidation to Uronic Acid Derivatives ***3a–d***

Under an argon atmosphere the benzoylated methyl pyranoside (0.4 mmol) was dissolved in MeCN/CCl_4_ (3 mL, 1/1 by vol.) and cooled to −10 °C. Then, a solution of CrO_3_-H_2_SO_4_ (200 μL, 0.48 mmol) was added dropwise over 5 min. The reaction mixture was stirred at the room temperature until full conversion as analysed by TLC (6–7 h). The mixture was then filtered, diluted with chloroform (15 mL), washed with saturated NaHCO_3_ (10 mL), NaCl (10 mL) and water (5 × 10 mL). Drying over MgSO_4_ and concentration under reduced pressure gave a syrup, which was crystallised from CHCl_3_/pentane to give a colourless solid.

#### 3.4.1. *Methyl 2,3,4-tri-*O*-Benzoyl-α-d-glucopyranuronic Acid* (**3a**)

*Methyl 2,3,4-tri-*O*-benzoyl-α-d-glucopyranoside* (**2a**, 800 mg, 1.58 mmol) [41] was reacted as described in Section 3.4 for 6 h. After work up and two crystallisations this gave 608 mg (1.17 mmol, 74%) of a colourless solid, mp. 117–118 °C, [α]${}_{D}^{20}$: = +82.0 (c 1.00, CHCl_3_). ^1^H-NMR (400 MHz, DMSO-*d_6_*) δ: 7.88–7.77 (m, 6H), 7.66–7.37 (m, 9H), 5.92 (t, *J* = 9.8, 1H, H-3), 5.65 (t, *J* = 9.8, 1H, H-4), 5.37 (dd, *J* = 10.2, 3.4, 1H, H-2), 5.30 (d, *J* = 3.4, 1H, H-1), 4.45 (d, 1H, *J* = 10.2, 1H, H-5), 3.46 (s, 3H). ^13^C-NMR (100 MHz, DMSO-*d_6_*) δ: 169.8 (C-6), 165.2, 164.8, 164.4, 134.1–128.2 (18C), 96.5 (C-1), 71.0 (C-2), 70.3 (C-3), 69.8 (C-4), 68.5 (C-5), 55.4 (OMe). HRMS (ESI): 521.1417 (calcd. 521.1442, M+H^+^). 

#### 3.4.2. *Methyl 2,3,4-tri-*O*-Benzoyl-α-d-galactopyranuronic Acid* (**3b**)

*Methyl 2,3,4-tri-*O*-benzoyl-α-d-galactopyranoside* (**2b**, 200 mg, 0.39 mmol) [41] was reacted as described in Section 3.4 for 6 h. After work up and two crystallisations this gave 154 mg (0.30 mmol, 77%) of a colourless solid, mp. 111–111 °C, [α]${}_{D}^{20}$: = +286.2 (c 1.00, CHCl_3_). ^1^H-NMR (400 MHz, DMSO-d_6_) δ: 7.88 (m, 4 H), 7.75–7.51 (m, 7H), 7.48–7.31 (m, 4H), 5.45 (dd, *J* = 10.3, 8.0, 1H, H-2), 5.99 (dd, 1H, *J* = 10.3, 3.7, H-4), 5.81 (dd, *J* = 10.3, 3.7, 1H, H-3), 5.45 (dd, *J* = 10.3, 8.0, 1H, H-2), 4.99 (m, 2H, H-1, H-5), 3.50 (s, 3H). ^13^C-NMR (100 MHz, CDCl_3_) δ: 168.0 (C-6), 165.1, 164.8, 164.6, 133.9-128.6 (18C), 96.9 (C-1), 70.2 (C-4), 68.4 (C-2), 68.12 (C-3), 68.09 (C-5), 55.5 (OMe). HRMS (ESI): 521.1454 (calcd. 521.1442, M+H^+^).

#### 3.4.3. *Methyl 2,3,4-tri-*O*-Benzoyl-β-d-galactopyranuronic Acid* (**3c**)

*Methyl 2,3,4-tri-*O*-benzoyl-β-d-galactopyranoside* (**2c**, 200 mg, 0.39 mmol) [41] was reacted as described in Section 3.4. After work up and two crystallisations this gave 161 mg (0.31 mmol, 79%) of a colourless solid, mp. 105–106 °C, [α]${}_{D}^{20}$: = +85.2 (c 1.00, CHCl_3_). ^1^H-NMR (400 MHz, DMSO-*d_6_*) δ: 7.93–7.86 (m, 4H), 7.75–7.44 (m, 9H), 7.34 (m, 2H), 6.08 (m, 1H, H-4), 5.87 (dd, *J* = 10.8, 3.3, 1H, H-3), 5.54 (dd, *J* = 10.8, 3.3, 1H, H-2), 5.36 (d, 1H, *J* = 3.3, 1H, H-1), 4.95 (m, 1H, H-5), 3.46 (s, 3H). ^13^C-NMR (100 MHz, DMSO-*d_6_*) δ: 167.6 (C-6), 164.8, 164.8, 164.6, 134.0-128.4 (18C), 100.4 (C-1), 71.6 (C-3), 71.2 (C-4), 69.7 (C-2), 65.5 (C-5), 56.6 (OMe). HRMS (ESI): 521.1442 (calcd. 521.1442, M+H^+^).

#### 3.4.4. *Methyl 2,3-di-*O*-Benzoyl-4-fluoro-α-d-glucopyranuronic Acid* (**3d**)

*Methyl 2,3-di-*O*-benzoyl-4-deoxy-4-fluoro-α-d-glucopyranoside* (**2d**, 800 mg, 1.98 mmol) was reacted as described in Section 3.4. After work up and two crystallisations this gave 653 mg (1.56 mmol, 79%) of a colourless solid, mp. 114–115 °C, [α]${}_{D}^{20}$: = +132.4 (c 1.00, CHCl_3_). ^1^H-NMR (400 MHz, DMSO-*d_6_*) δ: 7.93–7.86 (m, 4H), 7.75–7.44 (m, 9H), 7.34 (m, 2H), 6.08 (m, 1H, H-4), 5.87 (dd, *J* = 10.8, 3.3, 1H, H-3), 5.54 (dd, *J* = 10.8, 3.3, 1H, H-2), 5.36 (d, 1H, *J* = 3.3, 1H, H-1), 4.95 (m, 1H, H-5), 3.46 (s, 3H). ^13^C-NMR (100 MHz, DMSO-*d_6_*) δ: 172.7 (C-6), 166.0, 165.7, 134.2–128.6 (12C), 97.6 (C-1), 87.8 (d, *J* = 191.7, C-4), 71.1 (d, *J* = 7.1, C-2), 70.1 (d, *J* = 20.6, C-3), 68.3 (d, *J* = 24.6, C-5), 56.5 (OMe). ^19^F-NMR (470 MHz, DMSO-*d_6_*) δ: −196.3 (dd *J* = 50.5, 13.8). HRMS (ESI): 419.1131 (calcd. 419.1137, M+H^+^).

### 3.5. Methylation

The uronic acid (150 mg 0.28 mmol) in CH_2_Cl_2_/MeOH (4 mL, 3/1 by vol %) was reacted with trimethylsilyl diazomethane (100 μL, 2M in Et_2_O) under argon at 0 °C until full conversion (30–45 min.) as monitored by TLC. Then, the mixture was washed using sat. NaHCO_3_ (3 × 10 mL) and water (3 × 10 mL). After drying over MgSO_4_, and concentration at low pressure, the residue was purified by silica gel column chromatography (toluene/EtOAc, 9/1) followed by crystallisation from EtOH (twice).

#### 3.5.1. *Methyl (Methyl 2,3,4-tri-*O*-Benzoyl-α-d-glucoside)uronate* (**4a**)

*Methyl 2,3,4-tri-*O*-benzoyl-α-d-glucopyranuronic acid* (**3a**, 600 mg, 1.15 mmol) was treated as described in Section 4.5. This gave 468 mg (0.88 mmol, 77%) of a colourless solid, mp. 117–118 °C, [α]${}_{D}^{20}$: = +59.5 (c 1.00, CHCl_3_). ^1^H-NMR (400 MHz, CDCl_3_) δ: 8.00–7.86 (m, 6H), 7.55–7.30 (m, 9H), 6.19 (t, *J* = 9.9, 1H, H-3), 5.66 (t, *J* = 9.8. 1H, H-4), 5.32–5.34 (m, 2H, H-1+H-2), 4.58 (d, *J* = 10.1, 1H, H-5), 3.69 (s, 3H), 3.51 (s, 3H). ^13^C-NMR (100 MHz, CDCl_3_) δ: 168.3 (C-6), 165.9, 165.8, 165.5, 133.6-128.5 (18C), 97.6 (C-1), 71.66 (C-2), 70.4 (C-4), 69.8 (C-3), 68.7 (C-5), 56.4 (OMe), 53.0 (CO_2_Me). HRMS (ESI): 535.1588 (calcd. 535.1599, M+H^+^). 

#### 3.5.2. *Methyl (Methyl 2,3,4-tri-*O*-Benzoyl-α-d-galactoside)uronate* (**4b**) [38,42]

*Methyl 2,3,4-tri-*O*-benzoyl-α-d-galactopyranuronic acid* (**3b**, 150 mg, 0.29 mmol) was treated as described in Section 4.5. This gave 116 mg (0.22 mmol, 75%) of a colourless solid, mp. 126–127 °C, lit. [42] 134–135 °C, [α]${}_{D}^{20}$: = +198.5 (c 1.00, CHCl_3_), lit. [42] [α]${}_{D}^{20}$: = +238 (c 0.8, CHCl_3_). ^1^H-NMR corresponded well with that reported by Gill *et al*. [38] ^1^H-NMR (400 MHz, CDCl_3_) δ: 7.98 (4H, m), 7.79 (m, 2H), 7.44 (m, 9H), 6.21 (m, 1H, H-4), 5.98 (dd, *J* = 10.8, *J* = 3.4, 1H, H-3), 5.67 (dd, *J* = 10.8, 3.4, 1H, H-2), 5.43 (d, 1H, *J* = 3.4, 1H, H-1), 4.87 (m, 1H, H-5), 3.72 (s, 3H), 3.52 (s, 3H). ^13^C-NMR (100 MHz, CDCl_3_) δ: 167.6 (C-6), 166.1, 165.7, 165.4, 133.7–128.4 (18C), 98.1 (C-1), 70.2 (C-4), 69.0 (C-5), 68.9 (C-2), 68.0 (C-3), 56.6 (OMe), 52.9 (CO_2_Me). HRMS (ESI): 535.1599 (calcd. 535.1599, M+H^+^).

#### 3.5.3. *Methyl (Methyl 2,3,4-tri-*O*-Benzoyl-β-d-galactoside)uronate* (**4c**)

*Methyl 2,3,4-tri-*O*-benzoyl-β-d-galactopyranuronic acid* (**3c**, 150 mg, 0.29 mmol) was treated as described in Section 4.5. This gave 110 mg (0.21 mmol, 71%) of a colourless solid, mp. 112–113 °C, [α]${}_{D}^{20}$: = +173.1 (c 1.00, CHCl_3_). ^1^H-NMR (100 MHz, CDCl_3_) δ: 7.99 (m, 4H), 7.80 (m, 2H), 7.58–7.26 (m, 9H), 6.18 (dd, *J* = 3.4, 1.5, 1H, H-4), 5.80 (dd, *J* = 10.4, 8.0, 1H, H-2), 5.63 (dd, *J* = 10.4, 3.4, 1H, H-3), 4.76 (d, *J* = 8.0, 1H, H-1), 4.61 (d, *J* = 1.5, 1H, H-5), 3.72 (s, 3H), 3.64 (s, 3H). ^13^C-NMR (100 MHz, CDCl_3_) δ: 166.7 (C-6), 165.8, 165.4, 165.4, 133.7-128.5 (18C), 102.5 (C-1), 73.1 (C-5), 71.7 (C-3), 68.9 (C-2), 68.8 (C-4), 57.7 (OMe), 52.0 (CO_2_Me). HRMS (ESI): 535.1588 (calcd. 535.1599, M+H^+^).

#### 3.5.4. *Methyl (Methyl 2,3-di-*O*-Benzoyl-4-fluoro-α-d-glucoside)uronate* (**4d**)

*Methyl 2,3-di-*O*-benzoyl-4-fluoro-α-d-glucopyranuronic acid* (**3d**, 500 mg, 1.20 mmol) was treated as described in Section 4.5. This gave 421 mg (0.97 mmol, 81%) of a colourless solid, mp. 117–118 °C, [α]${}_{D}^{20}$: = +117.6 (c 1.00, CHCl_3_). ^1^H-NMR (400 MHz, CDCl_3_) δ: 7.98 (m, 4H), 7.51 (m, 2H), 7.38 (m, 4H), 6.08 (m, 1H, H-3), 5.23 (t, *J* = 3.4, 1H, H-1), 5.17 (ddd, *J* = 10.2, 3.5, 0.73, 1H, H-2), 4.92 (dt, *J* = 50.3, 9.2, 1H, H-4), 4.50 (m, 1H, H-5), 3.87 (s, 3H), 3.49 (s, 3H). ^13^C-NMR (100 MHz, CDCl_3_) δ: 168.3, (C-6), 165.9, 165.6, 133.7–128.5 (12C), 97.5 (C-1), 88.0 (d, *J* = 190.7, C-4), 71.2 (d, *J* = 8.1, C-2), 70.1 (d, *J* = 20.6, C-3), 68.4 (d, *J* = 24.9, C-5), 56.3 (OMe), 53.1 (CO_2_Me). ^19^F-NMR (470 MHz, CDCl_3_) δ: −196.85 (dd, *J* = 50.4, 13.7). HRMS (ESI): 433.1282 (calcd. 433.1293, M+H^+^).

### 3.6. Bromination Using HBr in AcOH

#### 3.6.1. *Methyl (2,3,4-tri-*O*-Benzoyl-α-d-glycopyranosyl bromide)uronate* (**5a**) [2,4,43]

Methyl (methyl 2,3,4-tri-*O*-benzoyl-α-d-glucoside)uronate (**4a**, 100 mg, 0.19 mmol) and a 33% hydrogen bromide/acetic acid solution (3 mL) was stirred under argon at the room temperature for 20 h. Then the solution was poured onto cold diethyl ether (15 mL), and the mixture was washed with saturated NaHCO_3_ (3 × 10 mL) and with cold water (3 × 10 mL). The ether phase was dried over MgSO_4_ and concentrated under reduced pressure. The resulting syrup was dissolved in benzene and crystallised by addition of pentane. After storage at 4 °C for 18 h, the solid formed were isolated by filtration. This gave 46 mg (0.08 mmol, 42%) of a colourless solid, mp. 143–144 °C, lit. [43] 146–148 °C, [43] [α]${}_{D}^{20}$: = +119.3 (c 1.00, CHCl_3_), lit [43]. +114.0 (c 1.31, CHCl_3_). ^1^H-NMR corresponded well with that reported previously [4]. ^1^H-NMR (400 MHz, CDCl_3_) δ: 7.93 (m, 6H), 7.59–7.28 (m, 9H), 6.89 (d, *J* = 4.0, 1H, H-1), 6.27 (t, *J* = 9.9, 1H, H-3), 5.73 (t, *J* = 9.9, 1H, H-4), 5.34 (dd, *J* = 9.9, 4.0, 1H, H-2), 4.86 (d, *J* = 10.3, 1H, H-5), 3.69 (s, 3H). ^13^C-NMR (100 MHz, CDCl_3_) δ: 166.9 (C-6), 165.6, 165.4, 165.3, 134.0–128.4 (18C), 85.8 (C-1), 72.7 (C-5), 71.2 (C-2), 70.0 (C-3), 69.2 (C-4), 53.3 (CO_2_Me). HRMS (ESI): 602.0838 (calcd. 602.0849, M+NH_4_^+^).

#### 3.6.2. *Methyl (2,3,4-tri-*O*-Benzoyl-α-d-galactopyranosyl bromide)uronate* (**5b**)

*Methyl (methyl 2,3,4-tri-*O*-benzoyl-α-d-galactoside)uronate* (**4b**) or *methyl (methyl 2,3,4-tri-*O*-benzoyl-α-d-galactoside)uronate* (**4c**, 100 mg, 0.19 mmol) was reacted as described in Section 3.6.1 to give 51 mg (0.09 mmol, 47%) of a colourless solid, mp. 126–127 °C, [α]${}_{D}^{20}$: = +212.7 (c 1.00, CHCl_3_). ^1^H-NMR (400 MHz, CDCl_3_) δ: 7.99 (m, 4H), 7.80 (m, 2H), 7.57–7.27 (m, 9H), 7.01 (d, *J* = 4.03, 1H, H-1), 6.29 (dd, *J* = 3.3, 1.3, 1H, H-4), 6.04 (dd, *J* = 10.4, 3.3, 1H, H-3), 5.66 (dd, *J* = 10.4, 4.0, 1H, H-2), 5.16 (d, *J* = 1.3, 1H, H-5), 3.74 (s, 3H). ^13^C-NMR (100 MHz, CDCl_3_) δ: 166.0 (C-6), 165.54, 165.50, 165.1, 134.0–128.5 (18C), 87.5 (C-1), 73.1 (C-5), 68.9 (C-4), 68.7 (C-3), 68.1 (C-2), 53.2 (CO_2_Me). HRMS (ESI): 602.0824 (calcd. 602.0849, M+NH_4_^+^).

### 3.7. Bromination Using ZnBr_2_/TMSBr

#### 3.7.1. *Methyl (2,3,4-tri-*O*-Benzoyl-α-d-glycopyranosyl bromide)uronate* (**5a**) Using Bromo-trimethylsilane/ZnBr_2_

To a stirred solution of methyl (methyl 2,3,4-tri-*O*-benzoyl-α-d-glucoside)uronate (**4a**) (500 mg, 0.94 mmol) and ZnBr_2_ (190 mg, 0.86 mmol) in dry CH_2_Cl_2_ (30 mL), bromotrimethylsilane (390 mg, 2.57 mmol) was added drop wise under argon and stirred for 24 h at the room temperature. The reaction mixture washed with saturated NaHCO_3_ (3 × 30 mL) and water (3 × 30 mL). Drying over MgSO_4_ and concentration under reduced pressure gave a syrup, which was dissolved in hot EtOH and stored at 4 °C for 18 h. This gave after filtration and drying 400 mg (0.69 mmol, 73%) of a white solid. The spectroscopic properties were identical to that described in Section 3.6.1.

#### 3.7.2. *Methyl (2,3-di-*O*-Benzoyl-4-deoxy-4-fluoro-α-d-glucosyl bromide)uronate* (**5d**)

The reaction was performed as described in Section 4.7.1 starting with methyl (methyl 2,3-di-*O*-benzoyl-4-deoxy-4-fluoro-α-d-glucoside)uronate (**4d**, 400 mg, 0.93 mmol). This gave after two crystallizations from EtOH a white solid 342 mg (0.71 mmol, 77%), mp. 106–107 °C, [α]${}_{D}^{20}$: = +120.6 (c 1.00, CHCl_3_). ^1^H-NMR (400 MHz, CDCl_3_) δ: 7.99 (4H), 7.54 (m, 2H), 7.40 (m, 4H), 6.77 (t, *J* = 3.5, 1H, H-1), 6.17 (m, 1H, H-3), 5.21 (ddd, *J* = 10.1, 4.2, 1.0, 1H, H-2), 5.00 (dt, *J* = 49.5, 9.2, 1H, H-4), 4.81 (m, 1H, 1H), 3.89 (s, 3H). ^13^C-NMR (100 MHz, CDCl_3_) δ: 166.9 (C-6), 165.4, 165.3, 134.1–128.3 (12C), 87.0 (d, *J* = 191.7, C-4), 85.4 (C-1), 72.4 (d, *J* = 25.9, C-5), 70.8 (d, *J* = 8.1, C-2), 70.3 (d, *J* = 20.9, C-3), 53.5 (CO_2_Me). ^19^F-NMR (470 MHz, CDCl_3_) δ: −197.42 (dd, *J* = 50.4, 13.7). HRMS (ESI): 498.0568 (calcd. 498.0569, M+NH_4_^+^).

### 3.8. Bromo-2,3,4-tri-O-acetyl-α-d-glucopyranuronic Acid Methyl Ester *(**8**)* *[30]*

Compound **8** was synthesized by a known procedure [30] starting from glucuronolactone (50.00 g, 284 mmol) to afford 30.77 g (77.4 mmol) of the title compound (27% total yield), mp. 101–102 °C, lit [30]. 102–105 °C. ^1^H-NMR (CDCl_3_) δ: 6.66 (d, *J* = 4.1, 1H, H-1), 5.60 (ap. t, *J* = 9.8, 1H, H-3), 5.23 (dd, *J* = 10.2, 9.8, 1H, H-4), 4.85 (dd, *J* = 9.9, 4.1, 1H, H-2), 4.57 (d, *J* = 10.2, H, H-5), 3.78 (3H, s, OCH_3_). 2.11 (s, 6H, CH_3_), 2.07 (s, 3H, CH_3_), 2.06 (s, 3H, CH_3_).

### 3.9. Lamotrigine Conjugates

#### 3.9.1. Lamotrigine Conjugate **10**

A mixture of lamotrigine (**9**, 30.0 mg, 0.12 mmol) and methyl(2,3,4-tri-*O*-benzoyl-α-d-glucopyranosyl bromide)uronate (**5a**, 100 mg, 0.17 mmol) were dissolved in dry nitromethane (3 mL). Anhydrous CdCO_3_ (20 mg, 0.12 mmol) was added and the mixture was heated at reflux. After 3 the reaction mixture was cooled, filtered and concentrated under reduced pressure to give syrup, which was dissolved in CHCl_3_ (10 mL) and washed by saturated NaHCO_3_ (10 mL) and water (3 × 10 mL). The organic layer was further dried over Na_2_SO_4_, evaporated and purified by preparative layer chromatography (PLC) plates eluted with CHCl_3_/EtOAc/MeOH (8/3/3). The resulting syrup was crystallised from CHCl_3_/pentane to give 46 mg (0.06 mmol, 50%) of **10** as a colourless amorphous solid. [α]${}_{D}^{20}$: = +75.2 (c 0.50, CHCl_3_). Main anomer: ^1^H-NMR (600 MHz, DMSO-d_6_) δ: 6.64 (bs, H-1), 6.19 (m, H-3), 6.06 (bs, H-2), 5.67 (m, H-4), 5.14 (d, *J* = 10.1, H-5), 3.57 (s, OMe). ^13^C-NMR (125 MHz, DMSO-d_6_) δ: 166.8 (C-6), 84.8 (C-1), 73.0 (C-5), 72.9 (C-3), 69.5 (C-4), 68.2 (C-2), 52.6 (OMe). HRMS (ESI): 759.1424 (calcd. 759.1488, M+H^+^). 

#### 3.9.2. Lamotrigine N-2-glucuronide **11**

Procedure A: A mixture of lamotrigine (50.0 mg, 0.197 mmol) and bromo-2,3,4-tri-*O*-acetyl-α-d-gluco pyranuronic acid methyl ester (50.0 mg, 0.125 mmol) was heated at 90 °C under argon. After 20 h a new portion of bromo-2,3,4-tri-*O*-acetyl-α-d-glucopyranuronic acid methyl ester (50.0 mg, 0.125 mmol) was added and the reaction continued for another 20 h. The melt was then cooled to room temperature, dissolved in methanol (15 mL) and filtered. The methyl ester and acetyl groups were hydrolyzed by addition of lithium hydroxide (0.1 M) until the pH reached 9.0 and stirring was continued at room temperature for 30 min. The alkaline solution was extracted with diethyl ether (3 × 70 mL) and the ether extract containing unrelated bromo-sugar and degradation products were discarded. The aqueous solution was diluted with glacial acetic acid until pH reached 6.0 and the mixture was then lyophilized at high vacuum. The solid material was dissolved in water, added to a Dowex 50W2-400 strong cation-exchange resin, and products eluted with 2 M aquas ammonia. Fractions with UV-response was pooled and lyophilized, before a final separation using preparative HPLC to give LTG-N2-glucuronide (6.2 mg, 0.014 mmol, 8% yield).

Procedure B. Lamotrigine (50 mg, 0.197 mmol) and bromo-2,3,4-tri-*O*-acetyl-α-d-glucopyranuronic acid methyl ester (100 mg, 0.250 mmol) was dissolved in dry nitromethane (4 mL). Anhydrous CdCO_3_ (50 mg, 0.290 mol) was added and the mixture was refluxed for 45 minutes under argon. The reaction mixture was poured into distilled water (10 mL) and filtered. Hydrolysis, extraction and final workup was performed as in procedure A to give LTG-N2-glucuronide (36.1 mg, 0.083 mmol, 42% yield. HRMS (ESI): 432.0472 (calcd. 432.0472, M^+^].

#### 3.9.3. Uronic Acid Conjugates **12** and **13**

A mixture of lamotrigine (**9**) (35 mg, 0.14 mmol) and 1-bromo-2,3-di-*O*-benzoyl-4-deoxo-4-fluoro-α-d-glucopyranuronate (**5d**, 90.0 mg, 0.19 mmol) were dissolved in dry nitromethane (3 mL). Anhydrous CdCO_3_ (20 mg, 0.12 mol) was added and the mixture was heated under reflux (75 °C). After 90 min the reaction mixture was cooled, filtered and concentrated under reduced pressure to give a syrup of **12** and **13**. The mixture was purified by preparative HPLC using a Varian Microsorb 100-8 C18 column (250 × 21.4 mm, flow rate 20 mL/min, injection volume 2 mL) with isocratic elution using a mixture of aq. ammonium acetate (20 mM)/MeOH (30/70, vol.%). Enriched fractions were lyophilized and further purified by preparative HPLC using a mixture of deionised water containing TFA (0.01%) and MeOH (30/70, vol.%) to give 14.5 mg (0.022 mmol, 16%) of **12** and 11 mg (0.017 mmol, 13%) of **13**.

Compound **12**: [α]${}_{D}^{20}$: = +88.0 (c 0.5, CHCl_3_). HRMS (ESI): 656.1115 (calcd. 656.1110, M+H^+^). Main anomer: ^1^H-NMR (600 MHz, DMSO-d_6_) δ: 6.33 (m, H-1), 6.01 (m, H-3), 6.00 (m, H-2), 5.30 (m, H-4), 5.00 (m, H-5), 3.79 (s, OMe). ^13^C-NMR (125 MHz, DMSO-d_6_) δ: 166.5 (C-6), 86.6 (d, *J* = 189.0, C-4), 85.3 (C-1), 73.2 (C-3), 72.9 (C-5), 67.5 (C-2), 52.8 (OMe). ^19^F-NMR (470 MHz, DMSO-d_6_) δ: −198.58 (m).

Compound **13**: [α]${}_{D}^{20}$: = +73.6 (c 0.5, CHCl_3_). HRMS (ESI): 636.1041 (calcd. 636.1047, M+H^+^). Main anomer: ^1^H-NMR (600 MHz, DMSO-d_6_) δ: 7.03 (bs, H-1), 6.26 (dd, *J* = 8.0, 2.7, H-3), 6.14 (d, *J* = 2.7, H-4), 5.96 (m, H-2), 3.76 (s, OMe). ^13^C-NMR (125 MHz, DMSO-d_6_) δ: 160.9 (C-6), 143.8 (C-5), 108.7 (C-4), 82.6 (C-1), 70.6 (C-3), 67.2 (C-2), 52.6 (OMe).

## 4. Conclusions

Benzoylated uronic acid building blocks have been prepared by a chemo-enzymatic approach using *Candida rugosa* for selective hydrolysis at C-6. The uronic acid derivatives might have different applications, one being the synthesis of glucuronides. Methyl (2,3,4-tri-*O*-benzoyl-α-d-glycopyranosyl bromide) uronate could be reacted with lamotrigine to give the N2-conjugate. The N2-glucuronide of lamotrigine was however most easily synthesised from the acetylated precursor using CdCO_3_ as base in nitromethane. This gave 41% yield and represents a major improvement to previously published synthetic procedure for producing quaternary *N*-glucuronides. Complete ^1^H- and ^13^C-NMR-assignments of the synthesized product have proven the existence of lamotrigine N2-glucuronide. Reaction of lamotrigine with the 4-deoxy-4-fluoro derivative **5d** was hampered by fluorine elimination as a side reaction, thus indicating the limitations of this building block in general.

## Figures and Tables

**Table 1:**
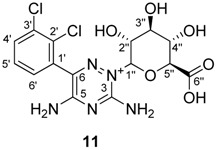
Proton and carbon NMR chemical shift assignments for Lamotrigine-N2-glucuronide.

Position	δ, ^1^H mult. (*J* in Hz)	δ, ^13^C	IP-COSY	NOESY	HMBC ^a^
3	-	155.3	-	-	-
5	-	156.0	-	-	-
6	-	140.8	-	-	-
1’	-	130.8	-	-	-
2’	-	132.1	-	-	-
3’	-	134.1	-	-	-
4’	7.79 m	133.9	5′ and/or 6′	5′ and/or 6’	2′, 6′, 3′ (w)
5’	7.50 m	129.5	^b^ 4′	^b^ 4′, 5-N**H**H (w), 5-NH**H** (w)	1′, 3′
6’	7.51 m	130.6			2′, 4′, 6 (w)
1”	5.53 br s	88.9	2″	3″, 5″, 3-NH_2_ (w)	3, 2″, 3″ (w)
2”	4.13 br t (9) ^c^	70.0	1″, 3″	4″, 3″ (w)	1″, 3″
3”	3.71 t (9.4) ^c^	76.7	2″, 4″	1″, 5″, 2″ (w), 4″ (w)	2″, 4″, 1″ (w)
4”	3.59 t (9.8) ^c^	71.7	3″, 5″	2″, 5″, 3″ (w)	3″, 5″, 6″
5”	4.02 d (10.1)	79.2	4”	1″, 3″, 4”	4″, 6″, 1″ (w), 3″ (w)
6”	-	175.1	-	-	-
3-NH_2_	8.21 br s	-	na	1″ (w)	na
5-N**H**H	8.00 br s	-	na	5-NH**H**, 5′ and/or 6′	5, 6
5-NH**H**	8.74 br s	-	na	5-N**H**H, 5′ and/or 6′	5 (w), 6 (w)

^a^ Correlations observed from proton to the listed carbon; ^b^ Due to overlap, COSY and NOESY correlations to 5’ and 6’ could not be distinguished; ^c^ Apparent triplets, but actually doublet of doublets. Individual coupling constants could not be resolved. s, Singlet; d, doublet; t, triplet; br, broad; w, weak; na, not available.
